# Effect of N-Acetylcysteine on Antioxidant Defense, Oxidative Modification, and Salivary Gland Function in a Rat Model of Insulin Resistance

**DOI:** 10.1155/2018/6581970

**Published:** 2018-01-30

**Authors:** Piotr Żukowski, Mateusz Maciejczyk, Jan Matczuk, Krzysztof Kurek, Danuta Waszkiel, Małgorzata Żendzian-Piotrowska, Anna Zalewska

**Affiliations:** ^1^Department of Restorative Dentistry, Croydon University Hospital, 530 London Road, Croydon, Surrey CR7 7YE, UK; ^2^Department of Physiology, Medical University of Bialystok, Mickiewicza 2c Str., 15-222 Bialystok, Poland; ^3^County Veterinary Inspection, Zwycięstwa 26B Str., 15-959 Bialystok, Poland; ^4^Department of Gastroenterology and Internal Medicine, Medical University of Bialystok, Sklodowskiej M.C. 24c Str., 15-274 Bialystok, Poland; ^5^Department of Conservative Dentistry, Medical University of Bialystok, Sklodowskiej M.C. 24a Str., 15-274 Bialystok, Poland; ^6^Department of Hygiene, Epidemiology and Ergonomics, Medical University of Bialystok, Mickiewicza 2c Str., 15-222 Bialystok, Poland

## Abstract

Oxidative stress plays a crucial role in the salivary gland dysfunction in insulin resistance (IR). It is not surprising that new substances are constantly being sought that will protect against the harmful effects of IR in the oral cavity environment. The purpose of this study was to evaluate the effect of N-acetylcysteine (NAC) on oxidative stress and secretory function of salivary glands in a rat model of insulin resistance. Rats were divided into 4 groups: C—normal diet, C + NAC—normal diet + NAC, HFD—high-fat diet, and HFD + NAC. We have demonstrated that NAC elevated enzymatic (superoxide dismutase, catalase, and peroxidase) and nonenzymatic antioxidants (reduced glutathione (GSH) and total antioxidant capacity (TAS)) in the parotid glands of HFD + NAC rats, while in the submandibular glands increased only GSH and TAS levels. NAC protects against oxidative damage only in the parotid glands and increased stimulated salivary secretion; however, it does not increase the protein secretion in the both salivary glands. Summarizing, NAC supplementation prevents the decrease of stimulated saliva secretion, seen in the HFD rats affected. NAC improves the antioxidative capacity of the both glands and protects against oxidative damage to the parotid glands of IR rats.

## 1. Introduction

Type 2 diabetes is a metabolic disease involving a genetic predisposition that leads to progressive relative insulin deficiency and resistance of peripheral tissues to its action [1]. It was estimated that the number of people with type 2 diabetes will increase from year to year, and by 2030, it will reach 366 million worldwide [2]. This dramatic rise is largely due to excessive weight, obesity, and lack of physical activity and less due to population growth, aging, and urbanization [2]. Moreover, according to the WHO, more than 80% of diagnosed cases are type 2 diabetes and this is primarily due to insulin resistance (IR) [3]. IR exists in prediabetic state, often over many years before the onset of symptoms of type 2 diabetes. The pathogenesis of IR is not fully understood; however, it appears that oxidative stress (OS) is one of the key mechanisms [4–6]. Oxidative stress is a situation in which increased production of reactive oxygen species (ROS) leads to cellular metabolism disorders and degradation of cellular structures, which results in oxidative organ damage and their dysfunction [7].

It has been proven that oxidative stress during high-fat diet-induced insulin resistance disrupts antioxidative systems of the parotid and submandibular salivary glands, leading to DNA, lipid, and protein oxidation and, in consequence, causing morphological changes of the glands, which is reflected in changes to the quality and quantity of saliva produced [8–10]. The data available confirms that salivary gland dysfunction manifests itself already at the IR stage and starting from this stage, diabetes can adversely affect oral health and the quality of life. It is well known that the changes in the salivary gland redox balance are responsible for salivary redox alterations [11, 12]. Considering the importance of the saliva in maintaining oral health, it is not surprising that the redox balance disturbances in the saliva dramatically increase the risk for development of oxidative stress-related oral maladies (dental caries, oral inflammatory infections like gingivitis, periodontitis, and oral mucosa ulceration, candidiasis, and burning mouth syndrome) [13–16]. These oxidative stress-related oral complications are diagnosed in almost all the patients affected by diabetes, even in well-controlled diabetes [17]. On the other hand, the presence of oral infections increases the risk and severity of diabetes. This relationship is due to the spread of the inflammatory mediators via bloodstream; moreover, the biological pathways that intensify diabetes are the same that intensify oral diseases [18, 19].

Synthetic antioxidants and nutrients with high antioxidant capacity showed the ability to prevent the oxidative damage and have been proposed as a unique therapeutic option for the treatment of the diabetes, IR, and their related complications [20–22]. N-Acetylcysteine (NAC) is an exogenous antioxidant, which works as a free radical scavenger. It is also a glutathione precursor regarded as one of the most important intracellular antioxidants. It has been proven that supplementation with N-acetylcysteine (NAC) activates antioxidant enzymes, prevents oxidative stress development, improves sensitivity towards insulin, and lowers insulin concentration in the serum of high-sugar diet-induced insulin resistant rats [20–23]. It was also shown that NAC supplementation prevents fructose-induced insulin resistance and hypertension [21] and inhibits development of diabetic peripheral neuropathies [24].

Scientific reports regarding the results of antioxidant supplementation on antioxidant defense, oxidative stress, oxidative damage, and function of the salivary glands in a rat model of insulin resistance are virtually nonexistent. Thus, the present study was undertaken to determine whether a chronic treatment of high-fat fed rats with N-acetylcysteine has a protective action against oxidative damage and prevents dysfunction of the salivary glands.

## 2. Materials and Methods

Male Wistar rats (cmdb outbred, 50–70 g, 4 weeks of age) after one week of adaptation were randomly assigned into four groups as follows: C—normal diet (*n* = 10), C + NAC—normal diet plus N-acetylcysteine (*n* = 10), HFD—high-fat fed (*n* = 10), and HFD + NAC—high-fat fed rats plus N-acetylcysteine (*n* = 10). For eight consecutive weeks of the experiment, the rats were fed high-fat diet (Research Diets Inc. cat. number D12492, composed of 59.8% fat, 20.1% protein, and 20.1% carbohydrates (kcal)) or normal diet (Agropol, Motycz, Poland, consisted of 10.3% fat, 24.2% protein, and 65.5% carbohydrates (kcal)). Every day, the rats from groups C + NAC and HFD + NAC were fed through an oral gavage N-acetylcysteine solution (500 mg/kg m.c; N-acetyl-L-cysteine A9165 Sigma). The dose of NAC was selected based on available literature. 500 mg/kg body weight of NAC is one of the more commonly used NAC doses that do not cause toxic symptoms in Wistar rats and have good antioxidants effects [25]. We decided on intragastric (i.g.) administration of NAC, because it is a dedicated route of administration to ensure that the animal receives a full dose of the drug in the experiment. Every morning, between the hours of 8 and 9, NAC was dissolved in saline immediately before administration and administered by gastric gavage in the amount of 2 mL/kg body weight by the one experienced person. Rats of the other groups received only intragastric saline solution in an amount of 2 mL/kg body weight.

The gastric administration of the liquid was always provided by the same 2 trained people (MM and JM). The rats were kept singly in standard cages and maintained at controlled temperatures (20-21°C), under standard condition of light from 6.00 a.m. to 6.00 p.m. and with free access to tap water.

Food consumption was measured once a week. Body weights were monitored every 2 days, and the amount of NAC was adjusted.

Rats were cared for in accordance with the principles and guidelines of the Institutional Committee for Ethics Use of Animals in the Medical University of Bialystok, Poland (protocol number 21/2017).

Eight weeks after being fed with the various diets, the rats were fasted for 12 hours and then anesthetized with phenobarbital (80 mg/kg of body weight). Rats were placed in a supine position on a heated (37°C) couch. Nonstimulated salivary secretion was measured for 15 minutes, using preweighted cotton balls inserted into the oral cavity underneath the tongue [26]. To evaluate the stimulated salivary secretory ability, rats were injected with pilocarpine hydrochloride (5 mg/kg BW, intraperitoneal, Sigma Chemical Co., St. Louis, MO, USA). Whole stimulated saliva was collected in a way analogous to the unstimulated secretion, 5 minutes after the pilocarpine administration, for 5 minutes [8]. The salivary flow was determined from the difference in the initial and final weight of the cotton balls. 1 mg of whole saliva was equal to 1 *μ*L [27].

Following that, tail blood glucose analysis was done (Accu Chek, Roche) followed by blood sampled from the abdominal aorta. The salivary glands were rapidly dissected out, weighed (laboratory weight KERN PLI 510-3M), immediately freeze-clamped with aluminium tongs, frozen in liquid nitrogen, and stored at −86°C for subsequent analysis. Blood was placed into glass tubes with heparin and spinned (5 min, 4°C, 3000 ×g, MPW 351, MPW Med Instruments, Warsaw, Poland) to obtain the plasma. For plasma samples, solution of BHT (10 *μ*L 0.5 M BHT in acetonitrile per 1 mL of the plasma, BHT; Sigma-Aldrich, Germany) and protease inhibitor (Complete Mini, Roche, France) were added. Plasma samples were precooled in liquid nitrogen and stored at −86°C.

The salivary gland fragments were immediately used to estimate the reduced glutathione, and the remaining salivary gland fragments were frozen similarly to plasma. On the day of performing analysis, the remaining salivary glands were divided into pieces and diluted (1 : 10) in ice cold PBS. The salivary gland remnants were homogenized with addition of the protease inhibitor (1 tablet/10 mL of the buffer) (Complete Mini, Roche, France) and the addition of antioxidant butyl-hydroxytoluene (10 *μ*L 0.5 M BHT in acetonitrile per 1 mL of the buffer) (BHT; Sigma-Aldrich, Germany), on ice (glass homogenizer; Omni TH, Omni International, Kennesaw, GA, USA), and after sonificated (1800 J/sample, 20 sec three times, on ice; ultrasonic cell disrupter, UP 400S, Hielscher, Teltow, Germany). The achieved salivary homogenates were centrifuged for 20 min, 4°C, 5000 ×g (MPW Med Instruments, Warsaw, Poland), and supernatants were analyzed the same day.

## 3. Biochemical Determinations

### 3.1. Enzymatic and Nonenzymatic Antioxidants

Catalase (CAT, EC 1.11.1.6) activity was analyzed in triplicate samples measuring the decrease in hydrogen peroxide (H_2_O_2_) consumption at 240 nm using Infinite M200 PRO Multimode Microplate Reader, Tecan [28].

Salivary peroxidase (Px, EC 1.11.1.7) activity was estimated colorimetrically measuring the absorbance changes at 412 nm in the reaction mixture containing DTNB (5,5′-dithiobis-2-nitrobenzoic acid), KI (potassium iodide), and H_2_O_2_ (hydrogen peroxide) [29].

Plasma glutathione peroxidase (GPx, EC 1.11.1.9) activity was measured colorimetrically at 340 nm estimating the conversion of NADPH to NADP+. One unit of GPx activity was defined as the amount of enzyme, which catalyzes the oxidation of 1 mmol NADPH per one minute [30].

Superoxide dismutase-1 (SOD-1, E.C. 1.15.1.1) activity was measured colorimetrically based on the epinephrine autoxidation at pH 10.2 in 37°C. One unit of SOD-1 activity was defined as the amount of enzyme, which inhibits oxidation of epinephrine by 50% [31].

The concentration of reduced glutathione (GSH) was analyzed colorimetrically by reaction with DTNB to give a complex that absorbs at wavelength 412 nm [32].

Total antioxidant status (TAS) was estimated in triplicate samples using a commercial kit according to the manufacturer's instructions (total antioxidant status (TAS) Randox (Crumlin, UK)). The ability of antioxidants contained in a sample to inhibit the formation of ABTS^•+^ (2,2′-azinobis-(3-ethylbenzothiazoline-6-sulfonate)) radical cation was measured colorimetrically at wavelength 600 nm [33].

All assays were performed in a duplicate samples, except for the CAT and TAS determination (see above), and standardized to mg of the total protein.

### 3.2. Oxidative Damage Products

The concentration of advanced oxidation protein products (AOPP) was analyzed colorimetrically at 340 nm measuring the total iodide ion-oxidizing capacity of the sample [34].

The content of advanced glycation end products (AGEs) were analyzed spectrofluorimetrically measuring the specific AGE fluorescence (350/440 nm). The final results were expressed as fluorescence/mg of the total protein [34].

The concentration of 8-hydroxy-2′-deoxyguanosine (8-OHdG) and 4-hydroxynonenal (4-HNE) protein adducts was estimated using ELISA method (8-hydroxy-2′-deoxyguanosine ELISA Kit, USCN, Life Science, Wuhan, China; OxiSelect™ HNE Adduct Competitive ELISA Kit, Cell Biolabs Inc. San Diego, CA, USA, resp.). 8-OHdG and 4-HNE determination was carried out according to the manufacturer's instructions, and color changes were measured at 450 nm.

Total oxidant status (TOS) was measured in triplicate samples using commercial kit PerOx (TOS/TOC) (Immundiagnostik AG, Bensheim, Germany) based on the oxidation of Fe^2+^ to Fe^3+^ in the presence of oxidants comprised in a sample [34]. Changes in absorbance were measured colorimetrically at wavelength 450 nm.

Oxidative stress index (OSI) was calculated according to the formula OSI = TOS/TAS × 100% [35].

The total protein was determined colorimetrically using BCA (bicinchoninic acid) method with BSA (bovine serum albumin) as a standard (Thermo Scientific Pierce BCA Protein Assay Kit, Rockford, IL, USA).

All assays were performed in duplicate samples, except for the TOS determination (see above), and converted to mg of the total protein.

The insulin concentration was determined by ELISA using commercially available kits (Shibayagi Co., Gunma, Japan; Cell Biolabs) following the attached instructions. The insulin sensitivity was calculated using the HOMA index of insulin resistance (HOMA-IR, homeostasis model assessment of insulin resistance) = fasting insulin (U/mL) × fasting glucose (mM)/22.5 [36].

## 4. Data Analysis

Statistical analysis was done using Statistica 10.0 (Statsoft, Cracow, Poland). The Kolmogorov-Smirnov test showed no normal distribution of the obtained results, which was the reason for using nonparametric methods. The Kruskal-Wallis test was performed to analyze quantitative values between the four groups followed by nonparametric multiple comparison test to assess differences among the specific groups. The data were explained as median (minimum–maximum). The Spearman correlation coefficient was used to study correlation between nonparametric variables. The statistical significance was defined as *p* ≤ 0.05.

## 5. Results

### 5.1. General Characteristics

At 8 weeks after treatment, body weights of the high-fat fed rats (HFD) were significantly higher compared to the control rats fed normal chow (*p* = 0.03). Chronic treatment with NAC prevented body weight gain, as body weights of HFD + NAC rats were significantly lower than rats fed only a high-fat diet (*p* = 0.03). HFD rats had higher glucose (*p* = 0.0002 and *p* = 0.03, resp.), insulin (*p* = 0.003 and *p* = 0.0009, resp.), plasma concentration, and HOMA-IR (*p* = 0.001 and *p* = 0.001, resp.) as compared to the control and HFD + NAC groups. HFD (*p* = 0.01) and HFD + NAC (*p* = 0.01) rats consumed significantly lesser chow than the control rats, while the energy intake was higher in HFD and HFD + NAC groups in relation to the control (*p* = 0.01 and *p* = 0.01, resp.) (Table 1).

The submandibular gland weights and the nonstimulated saliva secretions were similar among all the groups. The parotid gland weights of HFD rats were significantly higher as compared to the control rats (*p* = 0.005). NAC supplementation did not prevent the increase in weight of the parotid glands. These glands of HFD + NAC rats were significantly heavier than in the control group (*p* = 0.03). The HFD rats secreted less stimulated saliva than the rats given only normal chow (*p* = 0.003). Chronic treatment with NAC significantly increased stimulated saliva secretion in the HFD + NAC group in relation to the HFD group (*p* = 0.002) to a similar level as in the control rats given normal chow (Table 1).

### 5.2. The Plasma Oxidative Stress

The plasma total protein, 8-OHdG concentration, and GPx activity were comparable in all four groups (Table 2).

The HFD rats showed lower plasma SOD (*p* = 0.005) and CAT (*p* = 0.03) activities as well as TAS and GSH concentration (*p* = 0.006 and *p* = 0.03, resp.) as compared to the control rats fed normal chow. Chronic treatment with NAC significantly increased plasma SOD activity (*p* = 0.01) and GSH concentration (*p* = 0.008) as compared to HFD rats, to a similar level observed in the control rats, while significantly decreased plasma CAT activity (*p* = 0.01) in relation to the control rats. Chronic treatment with NAC significantly decreased plasma SOD activity and TAS concentration in the C + NAC group as compared to the control group (C), with *p* = 0.01 and *p* = 0.03, respectively (Table 2).

TOS (*p* = 0.02), AOPP (*p* = 0.008), 4-HNE protein adduct (*p* = 0.03) concentration, and OSI (*p* = 0.002) were increased in the plasma of the HFD group in relation to the control rats given normal chow (Table 2). NAC supplementation significantly decreased 4-HNE protein adduct (*p* = 0.01) and OSI (*p* = 0.03) in plasma of the HFD + NAC group as compared to the HFD rats, to a level observed in the control group (Table 2). Moreover, NAC prevented increase of the AOPP and TOS concentrations in the plasma of HFD + NAC rats, reaching values similar to the untreated control rats.

### 5.3. Parotid Glands

Effect of NAC supplementation on parotid glands SOD, CAT, and Px activities and GSH, TAS, TOS, OSI, 8-OHdG, AOPP, 4-HNE protein adduct, AGE, and total protein concentrations is given in Table 3.

There were no differences in AGE concentration in the parotid glands among the groups. HFD rats had lower parotid glands Px and SOD activities (*p* = 0.04 and *p* = 0.03, resp.) as well as GSH (*p* = 0.03) and TAS (*p* = 0.003) concentrations in relation to the control group fed normal chow. NAC supplementation significantly increased SOD, CAT, and Px activities (*p* = 0.04, *p* = 0.02, and *p* = 0.01, resp.) and GSH (*p* = 0.08) and TAS (*p* = 0.005) concentrations in the parotid glands of the HFD + NAC group as compared to the HFD group, to a similar level as in the control rats given normal chow. Moreover, NAC increased CAT activity in the parotid glands of the HFD + NAC groups in relation to the control (*p* = 0.02).

The HFD group showed higher TOS (*p* = 0.01), 8-OHdG (*p* = 0.08), AOPP (*p* = 0.02), 4-HNE protein adduct (*p* = 0.002) concentrations, and OSI (*p* = 0.003) in relation to the control group fed normal chow. NAC supplementation significantly decreased TOS (*p* = 0.01), 8-OHdG (*p* = 0.02), AOPP (*p* = 0.04), 4-HNE protein adduct (*p* = 0.0009), and OSI (*p* = 0.03) in the parotid glands of the HFD + NAC group as compared to the HFD group, to a similar level as in the untreated control rats.

Total protein in the parotid gland of HFD rats was decreased in relation to the control rats (*p* = 0.007). NAC supplementation did not prevent decrease of the protein content in the HFD group. Rats from the HFD + NAC had reduced protein concentration in the parotid gland as compared to the control rats (C) (*p* = 0.009).

### 5.4. Submandibular Gland

The effect of NAC supplementation on submandibular glands SOD, CAT, and Px activities and GSH, TAS, TOS, OSI, 8-OHdG, AOPP, 4-HNE protein adduct, AGE, and total protein concentrations is presented in Table 4.

The CAT and SOD activities and 8-OHdG concentration were similar between all groups. The HFD groups showed lower submandibular gland Px activity (*p* = 0.03), GSH (*p* = 0.04), and TAS (*p* = 0.03) concentrations as compared to the control. NAC supplementation increased the concentrations of GSH (*p* = 0.03) and TAS (*p* = 0.04) in the submandibular glands of the HFD + NAC group in comparison to the HFD rats; however, it did not prevent the decrease of Px activity. The specific activity of Px in the submandibular glands of HFD + NAC rats was significantly lower as compared to the control rats fed normal chow (*p* = 0.03).

The HFD group showed significantly higher submandibular gland: TOS (*p* = 0.005), AOPP (*p* = 0.004), 4-HNE protein adduct (*p* = 0.01), and AGE (*p* = 0.04) concentrations as well as OSI (*p* = 0.03) in relation to the rats fed normal chow (C). TOS (*p* = 0.09), 4-HNE protein adduct (*p* = 0.03), and AOPP (*p* = 0.02) concentration as well as OSI (*p* = 0.01) in the submandibular glands of HFD + NAC rats were significantly higher in relation to the control group given normal chow, and at the same time, significantly lower compared to the HFD rats (*p* = 0.04, *p* = 0.03, *p* = 0.03, and *p* = 0.04, resp.). NAC supplementation significantly increased 4-HNE protein adduct and AGE concentration in the submandibular glands of the C + NAC group (*p* = 0.003 and *p* = 0.02, resp.) in relations to the control.

Total protein in the submandibular gland of HFD rats was decreased in relation to the control rats (*p* = 0.02). NAC supplementation did not prevent decrease of the protein content in the HFD group. Rats from the HFD + NAC had reduced protein concentration in the submandibular gland as compared to the control rats (C) (*p* = 0.009).

### 5.5. Correlations

Positive correlation was shown between TAS and GSH concentrations in the submandibular gland (*p* = 0.03 and *r* = 0.52) as well as between 4-HNE protein adduct concentration in submandibular glands and plasma insulin (*p* = 0.02, *r* = 0.63) of HFD + NAC rats.

A positive correlation between SOD activity in the parotid glands and stimulated salivary flow (*p* = 0.01, *r* = 0.56) as well as negative relationship between SOD activity and plasma glucose concentration (*p* = 0.02, *r* = −0.47) of HFD + NAC rats were also noted.

## 6. Discussion

Saliva is secreted by the salivary glands, and it forms the liquid environment of the ecosystem of the mouth. Saliva determines homeostasis of the oral cavity due to the presence of organic and inorganic content, lubricating and buffering qualities, and specific and nonspecific defense mechanisms. In addition to the abovementioned host-protective properties, saliva is the first line of defense of the gastrointestinal tract against free radicals and their reactions with cellular components. The role of OS in the pathogenesis and development of salivary gland pathology in insulin resistance has been well established [8–11].

In the present study, we evaluated the antioxidant system and the parameters of oxidative stress in the salivary glands of high-fat fed rats supplemented with N-acetylcysteine (NAC). Basically, in the presence of NAC, the antioxidant barrier was restored and the oxidative stress was decreased in both parotid and submandibular glands.

A chronic high-fat diet was found to effectively induce obesity, hyperglycaemia, hyperinsulinemia, general oxidative stress, and insulin resistance [36–38]. It is not surprising, therefore, that the applied model of an 8-week high-fat diet resulted in a shift of the oxidative/antioxidant plasma balance towards the oxidative status as well as a significant increase in the body weight of rats and decreased whole body insulin sensitivity assessed by a significantly higher medians of insulin and glucose in the blood and the HOMA-IR index as compared to rats fed a standard diet [36–38].

Our results are also consistent with the facts that NAC affects glucose metabolism and has a potential effect as a therapeutic agent against the onset of insulin resistance, oxidative stress, and its complications [20–22]. During the eight weeks of the experiment, NAC supplementation prevents the onset of plasma OS (Table 2), reduced hyperglycaemia and hyperinsulinemia as well as HOMA-IR compared to the rats given a high-fat diet only, to the level observed in the control group given normal chow (Table 1). Despite similar caloric intake, HFD + NAC rats showed lower final body weight compared to HFD rats, and this was in agreement with Diniz et al. [39]. The reason for reduced body weight of HFD + NAC rats in relation to the HFD group was not assessed in our study, but previous study suggested that reduced body weight was caused by a NAC-induced decrease in intestinal absorption of the ingested chow [39].

The major body site for producing superoxide anion is the mitochondria, which is an organelle-processing chemical energy contained in the energy substrates (glucose and fatty acids) into the energy of anhydride pyrophosphate bonds of the ATP (adenosine triphosphate). Under a balanced diet, electrons are passed through a mitochondrial electron transport chain in order to reduce molecular oxygen to water. However, 1–3% of all electrons “escape” from this chain resulting in the formation of small quantities of superoxide anions, which are easily converted into hydrogen peroxide (H_2_O_2_) by superoxide dismutases (SOD). SOD works with other enzymes (peroxidase (Px), glutathione peroxidase (GPx), and catalase (CAT)) and nonenzymatic antioxidants (reduced glutathione (GSH)) that remove H_2_O_2_ and other reactive oxygen and nitrogen species (RNS), which allows the redox balance or the balance between ROS/RNS rates of production and their rates of elimination to be maintained [40]. In the case of a high-fat diet, the excess fatty acid supply in the respiratory chain increases dramatically, which results in the formation of excessive quantities of superoxide anions and subsequently other ROS and RNS [41]. The unbalanced reactive oxygen and nitrogen species during deficiency of antioxidant mechanisms can lead to development of oxidative stress and oxidative damage of cellular structures and dysfunction of cellular metabolism and its regulation [42]. Increased ROS can damage all cellular components. Therefore, to assess the presence, degree, and prognosis of OS, there are several markers of oxidative damage. The most common assessments to evaluate oxidative damage are 4-HNE protein adduct (for the lipids), advanced oxidation protein product (AOPP) (for the proteins), and 8-hydroxy-d-guanine-8-OHdG (for the DNA) [7].

Our results confirmed previous data showing that high-fat diet-induced insulin resistance increased the parotid and submandibular gland oxidative damage and decreased the antioxidant barrier of both types of glands as compared to the control. The parotid glands were more susceptible than the submandibular glands to oxidant attack generated in the course of high-fat diet-induced IR which we observed as a greater intensity and variety of oxidative damage, as already documented [8–11]. High-fat feeding-induced insulin resistance impaired stimulated salivary flow and affected the function of both glands in the course of protein synthesis/secretion, which was assessed as a significant decrease in the total protein concentration in both glands of the HFD rats in relation to the control and was in agreement with Kołodziej et al. [8].

The effect of NAC supplementation on the salivary glands of rats fed a high-fat diet is novel. Furthermore, this is the first report of an administration of any antioxidant agent aiming at protecting the salivary glands against OS in the course of high-fat feeding.

Administration of NAC has been reported to be beneficial in IR, diabetes, and their complications [20–22]. NAC is regarded as an antioxidant that acts either directly or by increasing intracellular GSH level [43]. Our results showed that NAC supplementation normalizes the antioxidant barrier in both the salivary glands of the high-fat fed rats (HFD + NAC rats), in comparison to the HFD group of rats. This is indicated by a significant TAS increase. TAS is one of the main parameters describing the antioxidant barrier; TAS is the sum of all antioxidants both in the parotid and in the submandibular glands. However, it is worth noticing that we observed a higher percentage TAS increase in the parotid (↑ 37%) than in the submandibular glands (↑ 8%) in the HFD + NAC versus HFD groups. In the submandibular glands of HFD + NAC rats, we observed only a significant increase of glutathione. Moreover, a positive correlation between TAS and GSH suggests that NAC normalizes the submandibular antioxidant barrier mainly by an increase of glutathione concentration. It should be, however, underlined that in the salivary antioxidant system, Px is by far the most important antioxidant, the only one synthesized in the salivary glands. Other salivary enzymes like SOD, CAT, and GPx or nonenzymatic antioxidants come mainly from the blood vessels and have only marginal antioxidant significance [44]. Persistent drop in Px activity in the submandibular salivary gland, despite NAC supplementation, is a proof that the antioxidant barrier is deficient in combating ROS. On the contrary, in the parotid glands of the HFD + NAC group, the increase of total antioxidant barrier was achieved not only by essential increase of GSH but also by essential increases of SOD, Px, and CAT activities, which indicates an activation of the enzymatic antioxidant barrier. The increase of the abovementioned antioxidant enzymes may be related to previously cited NAC-suppressed NF-*κ*B (nuclear factor kappa B) activation and upregulation of the gene expression of these enzymes [45, 46]. High-fat feeding has been shown to be associated with reduced mRNA expression of SOD1, CAT, and GPx in adipose tissue [47]. On the other hand, the negative correlation between SOD activity and blood glucose concentration in the parotid gland of HFD + NAC rats suggests that hyperglycaemia could be a significant factor in the downregulation of enzymatic antioxidant capacity, which was observed earlier [48].

The difference in parotid and submandibular antioxidant defense mechanisms is not understood. It may come from the fact that the parotid glands are the main source of antioxidants while the mandibular glands' share of the total salivary antioxidant capacity is insignificant [10, 44]. The parotid glands are also capable of more antioxidant response to counterbalance ROS, and presumably, the addition of exogenous antioxidant increases their antioxidant potential and allows the parotid glands to counteract the excess of ROS effectively. Essential reduction of the concentration of TOS (↓ 45%), 8-OHdG (↓ 20%), AOPP (↓ 49.7%), 4-HNE-protein adduct (↓ 97%), and OSI (↓ 70%) was observed in the parotid gland of the HFD + NAC group versus HFD rats. Furthermore, concentrations of the abovementioned parameters of oxidative stress in the parotid glands of the HFD + NAC group did not differ from concentrations in the parotid glands of the rats fed a standard diet, which proves that the 8-week NAC treatment prevents oxidative damage and allows maintaining the redox balance in these glands. As the salivary antioxidant capacity is determined mainly by the antioxidant efficiency of parotid glands, the results obtained are of great importance. It points to the fact that the oral cavity will be sufficiently protected against high-fat fed-induced enhanced ROS generation.

In the case of the submandibular gland, we observed a significantly higher OSI index (the oxidative stress index which evaluates relationship between the total oxidant status (TOS) and total antioxidant status (TAS)) and significantly higher TOS versus control group. NAC supplementation could not prevent oxidative damage in these glands, indicated by increased concentration of AOPP and 4-HNE protein adduct versus control glands. It should be stressed that our study showed some degree of a protective effect of NAC in decreasing oxidative stress in the submandibular glands of rats given high-fat chow. The NAC protective action was seen in significantly lower concentrations of TOS (↓ 23%), OSI (↓ 61%), AOPP (↓ 18%), and 4-HNE protein adduct (↓ 44%) in the submandibular glands of the HFD + NAC versus HFD rats. Moreover, NAC prevented an increase of AGE concentration in the submandibular glands of the HFD + NAC rats. AGE concentration in the submandibular glands of the HFD + NAC was similar to the control rats.

Since NAC can act as a prooxidants, it has been suggested to avoid its prophylactic administration in situations that are not accompanied by oxidative stress [49, 50], which is in accordance with our results. A significant increase in 4-HNE protein adduct and AGE concentration in the submandibular gland and a significant decrease in SOD activity and TAS concentration in plasma of C + NAC rats versus control confirm that in the healthy control, NAC raises the prooxidant milieu rather than prevents oxidative stress.

No correlation between antioxidant/oxidant products in the salivary glands and plasma was observed in any of the groups. This indicates that the redox balance in the salivary glands is independent from blood plasma and that the antioxidant mechanisms of the salivary glands and the intensity of oxidative stress are a result of actions within the salivary glands, not diffusion from the blood vessels. However, we noticed a positive correlation between insulin concentration and concentration of 4-HNE protein adduct in submandibular glands of the HFD + NAC group. This result is in agreement with the data that the hyperinsulinemia leads to insufficient scavenging of hydrogen peroxide. Hydrogen peroxide undergoes Fenton reaction to generate ·OH which is implicated in the onset and propagation of lipid peroxidation [51].

It should be emphasized that in humans, the parotid gland produces mainly stimulated saliva, while submandibular/sublingual salivary glands secrete without stimulation. Any quantitative or qualitative changes in stimulated saliva reflect solely parotid gland dysfunction. We noted that chronic treatment with NAC prevented dysfunction of parotid acinar machinery involved in stimulated saliva secretion, seen in the HFD rats affected. It should be underlined that high-fat feeding did not induce impairment in the nonstimulated saliva secretion, so it is not surprising that any protective effect of NAC was not observed. It is considered that disrupted secretion of stimulated saliva in patients with IR or diabetes is a result of neurotransmission disruption and has a multifactorial etiology that involves remodeling of the extracellular matrix, inflammation, degradation of acinar cells, and oxidative stress. NAC has an antioxidant, but also the anti-inflammatory and antiapoptotic properties. Moreover, NAC positively affects neurotransmission and inhibits the onset of diabetic peripheral neuropathies [24]. All these NAC properties presumably could facilitate function and communication of residual of the neural and acinar cells, which prevent reduced ability of the parotid glands to respond to external stimuli.

On the other hand, there is a positive correlation between SOD activity in the parotid glands of the HFD + NAC rats and stimulated saliva secretion rate, which suggests another mechanism preventing reduction of salivary secretion. It was shown that SOD activates muscarinic receptor M1 inducing extracellular signaling-regulated protein kinase and modulating synaptic transmission in neuroblastoma SK-N-BE cells [52]. Receptor M1 activation in the salivary glands stimulates water flow into glandular cells and increases secretion of stimulated saliva.

In contrast to saliva secretion, NAC did not prevent disruption to the protein synthesis mechanism in parotid and submandibular glands, which is observed as a significant decrease in the total protein content in both salivary glands of the HFD + NAC rats versus control. It therefore appears that factors other than OS, inflammation, neuropathies, or other NAC-related mechanisms influenced the reduction in the total protein content in both glands. It was documented that a reduced protein concentration in the salivary glands may be due to reduced food intake. Evidence showed that decreased food intake causes alterations in protein synthesis or their exocytosis, which was indicated as a higher storage of granules/vehicles with secretory proteins and decreased protein concentrations in salivary glands [53]. On the other hand, the NAC dose used by us, however useful for the redox balance and physiological saliva secretion, may not be sufficient to prevent protein synthesis disruption in the salivary glands of HFD-fed rats.

Considering the protective effect of NAC against the salivary dysfunction induced by high-fat feeding, one should keep in mind that this effect could be secondary to the systemic effect of NAC (preventing of insulin resistance). Only kinetic studies could address this problem. However, Murley et al. [54] showed that thiol-containing drugs, which have some indications of NAC molecular mechanisms, by NF-*κ*B pathway influenced the expression of antioxidant enzymes in SA-NH tumor cells.

## 7. Conclusions

1. In summary, our results showed that chronic treatment of high-fat fed rats with NAC prevents the decrease of stimulated saliva secretion, seen in the HFD rats affected. Reduced protein concentration can be observed in parotid and submandibular glands of high-fat fed rats despite the chronic NAC treatment.
2. Supplementation of NAC was effective in increasing the antioxidant barrier of both glands.
3. NAC supplementation offered a protection to parotid glands against oxidative stress, while the severity and extent of oxidative damage to the submandibular glands of HFD + NAC rats were only reduced by the NAC treatment.

## Figures and Tables

**Table 1:** Effect of NAC supplementation on body weight, fasting plasma glucose, insulin concentration HOMA-IR, food intake, unstimulated and stimulated saliva secretion, protein concentration, and salivary gland weight.

	C (*n* = 10) M (min–max)	C + NAC (*n* = 10) M (min–max)	HFD (*n* = 10) M (min–max)	HFD + NAC (*n* = 10) M (min–max)
Final body weight (g)	282 (260–298)	286 (261–319)	345 (326–364)^∗∗^	305 (250–304)^∗^
Glucose (mg/dL)	89 (86–93)	95 (92–97)	145 (130–161)^∗∗^	95 (87–99)^∗^
Insulin (mIU/mL)	79.70 (29.3–92.36)	80.48 (42.22–96.96)	166.42 (158.2–176.88)^∗∗^	61.25 (39.55–81.18)^∗^
HOMA-IR	3.64 (1.87–6.15)	5.67 (2.45–6.25)	19.60 (15.45–21.05)^∗∗^	4.33 (2.54–5.10)^∗^
Food intake (mg/day/rat)	17.68 (16.43–18.45)	16.92 (15.98–19.23)	11.82 (10.01–13.63)^∗∗^	12.25 (10.21–13.89)^∗∗^
Energy from chow (MJ/day/rat)	0.16 (0.15–0.18)	0.15 (0.14–0.19)	0.26 (0.22–0.29)^∗∗^	0.27 (0.22–0.30)^∗∗^
NS (*μ*L/min)	0.43 (0.41–0.58)	0.43 (0.40–0.51)	0.40 (0.25–0.55)	0.46 (0.30–0.50)
SS (*μ*L/min)	109.67 (104.58–121.37)	91.84 (82.20–102.8)	68.60 (56.38–70.40)^∗∗^	115.25 (96.67–152.63)^∗∗^
Parotid weight (mg)	76.22 (74.10–79.90)	79.52 (72.90–87.60)	99.46 (91.00–112.10)^∗∗^	92.55 (81.10–112.20)^∗∗^
Submandibular weight (mg)	211.64 (184.10–240.20)	208.52 (192.60–222.90)	250.04 (216.40–273.00)	264.38 (221.90–304.20)

C: control rats; C + NAC: control rats + N-acetylcysteine; HFD: high-fat fed rats; HFD + NAC: high-fat fed rats + N-acetylcysteine; HOMA-IR: homeostasis model assessment of insulin resistance; NS: nonstimulated saliva secretion; SS: stimulated saliva secretion: M (min–max): median (minimum–maximum). ^∗^*p* < 0.05 HFD and HFD + NAC; ^∗∗^*p* < 0.05 HFD and C; HFD + NAC and C.

**Table 2:** Effect of NAC supplementation on plasma SOD, CAT, and GPx activities and GSH, TAS, TOS, OSI, 8-OHdG, AOPP, 4-HNE protein adduct, AGE, and total protein concentrations.

Plasma	C (*n* = 10) M (min–max)	C + NAC (*n* = 10) M (min–max)	HFD (*n* = 10) M (min–max)	HFD + NAC (*n* = 10) M (min–max)
SOD (mU/mg of protein)	0.76 (0.65–0.88)	0.32 (0.20–0.55)^∗∗^	0.27 (0.22–0.33)^∗∗^	0.59 (0.48–0.73)^∗^
CAT (nmol H_2_O_2_/min/mg of protein)	1.53 (1.00–1.78)	0.78 (0.47–1.28)	0.73 (0.61–0.96)^∗∗^	0.66 (0.43–0.77)^∗∗^
GPx (*μ*U/mg of protein)	32.85 (17.65–39.99)	27.39 (18.25–36.32)	34.45 (24.97–50.43)	46.88 (33.14–53.10)
GSH (ng/mg of protein)	0.72 (0.55–0.92)	0.67 (0.46–0.94)	0.28 (0.18–0.47)^∗∗^	0.91 (0.84–1.05)^∗^
TAS (*μ*mol/mg of protein)	0.62 (0.56–0.65)	0.42 (0.35–0.53)^∗∗^	0.38 (0.34–0.41)^∗∗^	0.48 (0.38–0.58)
TOS (*μ*mol/mg of protein)	1.45 (0.77–1.86)	2.64 (1.04–4.16)	3.52 (2.95–4.24)^∗∗^	2.08 (0.92–3.32)
OSI	227.85 (122.68–296.46)	604.77 (286.40–901.52)	927.7 (781.51–1211.9)^∗∗^	446.87 (177.89–726.32)^∗^
8-OHdG (pg/mg of protein)	0.02 (0.01–0.04)	0.19 (0.01–0.44)	0.12 (0.02–0.21)	0.18 (0.02–0.34)
AOPP (nmol/mg of protein)	1.55 (1.13–1.81)	2.67 (1.59–5.36)	3.81 (2.85–4.61)^∗∗^	2.24 (1.54–2.57)
4-HNE protein adduct (*μ*g/mg of protein)	30.42 (14.33–50.44)	36.29 (24.47–53.98)	70.47 (66.52–78.64)^∗∗^	19.24 (2.46–42.48)^∗^
AGE (fluorescence/mg of protein)	0.89 (0.76–0.98)	1.37 (0.86–2.61)	1.41 (1.04–1.94)	1.16 (0.73–1.15)
Protein (*μ*g/mL)	4551 (4103–4964)	4423.9 (3588.6–5027.4)	4271.0 (3471.3–4848.8)	3886.2 (3432.0–5027.4)

C: control rats; C + NAC: control rats + N-acetylcysteine; HFD: high-fat fed rats; HFD + NAC: high-fat fed rats + N-acetylcysteine; M: median: min: minimum; max: maximum; SOD: superoxide dismutase; CAT: catalase, GPx: glutathione peroxidase; GSH: reduced glutathione; TAS: total antioxidant status; TOS: total oxidant status; OSI: oxidative status index; 8-OHdG: 8-hydroxy-d-guanosine; AOPP: advanced oxidation protein products; 4-HNE protein adduct: 4 hydroxynonenal protein adduct; AGE: advanced glycation end products. ^∗^*p* < 0.05 HFD and HFD + NAC; ^∗∗^*p* < 0.05 C + NAC and C; HFD and C; HFD + NAC and C.

**Table 3:** Effect of NAC supplementation on parotid glands SOD, CAT, and Px activities and GSH, TAS, TOS, OSI, 8-OHdG, AOPP, 4-HNE protein adduct, AGE, and total protein concentrations.

Parotid glands	C (*n* = 10) M (min–max)	C + NAC (*n* = 10) M (min–max)	HFD (*n* = 10) M (min–max)	HFD + NAC (*n* = 10) M (min–max)
SOD (mU/mg of protein)	0.62 (0.44–1.17)	0.37 (0.33–0.42)	0.29 (0.18–0.31)^∗∗^	0.57 (0.36–0.68)^∗^
CAT (nmol H_2_O_2_/min/mg of protein)	0.81 (0.59–0.97)	0.84 (0.53–1.13)	0.76 (0.09–1.09)	1.26 (1.2–1.74)^∗^^,^^∗∗^
Px (*μ*U/mg of protein)	55.7 (51.4–59.5)	34.5 (25.5–60.6)	37.4 (33.5–39.6)^∗∗^	52.9 (46.7–55.9)^∗^
GSH (ng/mg of protein)	0.94 (0.95–0.99)	1.12 (0.70–1.55)	0.73 (0.52–0.88)^∗∗^	1.18 (0.86–1.73)^∗^
TAS (*μ*mol/mg of protein)	0.67 (0.65–0.99)	0.65 (0.53–0.82)	0.32 (0.27–0.49)^∗∗^	0.92 (0.66–1.13)^∗^
TOS (*μ*mol/mg of protein)	1.30 (0.76–1.78)	0.69 (0.36–0.98)	2.24 (1.98–2.54)^∗∗^	1.25 (0.47–1.67)^∗^
OSI	192.9 (116.0–258.0)	114.3 (44.0–172.0)	536.9 (403.6–620.6)^∗∗^	165.8 (68.0–366.3)^∗^
8-OHdG (pg/mg of protein)	1.47 (1.31–1.58)	1.19 (0.89–1.39)	1.91 (1.80–1.99)^∗∗^	1.53 (1.16–1.57)^∗^
AOPP (nmol/mg of protein)	10.0 (6.2–12.4)	9.6 (1.7–12.8)	23.4 (18.0–34.1)^∗∗^	11.64 (7.90–14.6)^∗^
4-HNE protein adduct (*μ*g/mg of protein)	10.70 (2.16–18.9)	5.70 (1.59–10.54)	50.7 (30.4–57.3)^∗∗^	1.78 (0.34–2.91)^∗^
AGE (fluorescence/mg of protein)	0.41 (0.36–0.46)	0.38 (0.28–0.53)	0.41 (0.35–0.52)	0.48 (0.44–0.52)
Protein (*μ*g/mL)	4419 (4054–4889)	3654 (3348–4031)	3324 (3000–3828)^∗∗^	3292.52 (2930.30–3457.30)^∗∗^

C: control rats; C + NAC: control rats + N-acetylcysteine; HFD: high-fat fed rats; HFD + NAC: high-fat fed rats + N-acetylcysteine; M: median; min: minimum; max: maximum; SOD: superoxide dismutase; CAT: catalase; Px: peroxidase; GSH: reduced glutathione; TAS: total antioxidant status; TOS: total oxidant status; OSI: oxidative status index; 8-OHdG: 8-hydroxy-d-guanosine; AOPP: advanced oxidation protein products; 4-HNE protein adduct: 4 hydroxynonenal protein adduct; AGE: advanced glycation end products. ^∗^*p* < 0.05 HFD and HFD + NAC; ^∗∗^*p* < 0.05 C + NAC and C; HFD and C; HFD + NAC and C.

**Table 4:** Effect of NAC supplementation on submandibular glands SOD, CAT, and Px activities and GSH, TAS, TOS, OSI, 8-OHdG, AOPP, 4-HNE protein adduct, AGE, and total protein concentrations.

Submandibular glands	C (*n* = 10) M (min–max)	C + NAC (*n* = 10) M (min–max)	HFD (*n* = 10) M (min–max)	HFD + NAC (*n* = 10) M (min–max)
SOD (mU/mg of protein)	0.33 (0.12–0.48)	0.36 (0.33–0.41)	0.33 (0.23–0.39)	0.37 (0.24–0.49)
CAT (nmol H_2_O_2_/min/mg of protein)	1.18 (0.98–1.36)	1.02 (0.76–1.27)	1.1 (0.8–1.4)	1.34 (1.11–1.65)
Px (*μ*U/mg of protein)	48.36 (44.53–53.91)	38.8 (28.1–56.1)	32.76 (22.11–40.04)^∗∗^	31.78 (29.24–35.30)^∗∗^
GSH (ng/mg of protein)	1.09 (0.95–1.18)	1.31 (1.05–1.64)	0.56 (0.16–0.76)^∗∗^	0.95 (0.83–1.21)^∗^
TAS (*μ*mol/mg of protein)	0.56 (0.43–0.73)	0.63 (0.44–0.81)	0.35 (0.22–0.46)^∗∗^	0.61 (0.55–0.78)^∗^
TOS (*μ*mol/mg of protein)	0.61 (0.37–0.79)	0.58 (0.34–0.87)	1.6 (1.4–1.9)^∗∗^	1.24 (1.05–1.35)^∗^^,^^∗∗^
OSI	118.47 (50.47–169.84)	89.92 (58.09–114.92)	471.10 (326.46–621.83)^∗∗^	187.2 (179.4–280.7)^∗^^,^^∗∗^
8-OHdG (pg/mg of protein)	1.23 (1.02–1.41)	1.29 (1.11–1.47)	1.31 (1.01–1.87)	1.42 (1.36–1.49)
AOPP (nmol/mg of protein)	9.35 (7.90–11.01)	9.81 (9.32–12.57)	18.85 (17.69–23.31)^∗∗^	15.47 (12.74–16.97)^∗^^,^^∗∗^
4-HNE protein adduct (*μ*g/mg of protein)	7.29 (3.74–10.61)	32.82 (9.99–46.88)^∗∗^	37.88 (27.41–45.84)^∗∗^	21.27 (13.24–26.02)^∗^^,^^∗∗^
AGE (fluorescence/mg of protein)	0.26 (0.21–0.37)	0.5 (0.45–0.52)^∗∗^	0.41 (0.39–0.45)^∗∗^	0.32 (0.20–0.55)
Protein (*μ*g/mL)	3804 (3567–3933)	3548 (3413–3673)	3210 (3080–3387)^∗∗^	3282 (3160–3423)^∗∗^

C: control rats; C + NAC: control rats + N-acetylcysteine; HFD: high-fat fed rats; HFD + NAC: high-fat fed rats + N-acetylcysteine; M: median; min: minimum; max: maximum; SOD: superoxide dismutase; CAT: catalase; Px: peroxidase; GSH: reduced glutathione; TAS: total antioxidant status; TOS: total oxidant status; OSI: oxidative status index; 8-OHdG: 8-hydroxy-d-guanosine; AOPP: advanced oxidation protein products; 4-HNE protein adduct: 4 hydroxynonenal protein adduct; AGE: advanced glycation end products. ^∗^*p* < 0.05 HFD and HFD + NAC; ^∗∗^*p* < 0.05 C + NAC and C; HFD and C; HFD + NAC and C.
